# Pharmacokinetics, safety, and efficacy of a single co-administered dose of diethylcarbamazine, albendazole and ivermectin in adults with and without *Wuchereria bancrofti* infection in Côte d’Ivoire

**DOI:** 10.1371/journal.pntd.0007325

**Published:** 2019-05-20

**Authors:** Constant Edi, Catherine M. Bjerum, Allassane F. Ouattara, Yashpal S. Chhonker, Louis K. Penali, Aboulaye Méité, Benjamin G. Koudou, Gary J. Weil, Christopher L. King, Daryl J. Murry

**Affiliations:** 1 Centre Suisse de Recherche Scientifique en Côte d’Ivoire, Côte d’Ivoire; 2 Center for Global Health and Diseases, Case Western Reserve University School of Medicine, Cleveland, OH, United States of America; 3 Université Nangui Abrogoua, Côte d’Ivoire; 4 Dept of Pharmacy Practice, University of Nebraska Medical Center, Omaha, NE, United States of America; 5 Institut Pasteur de Côte d’Ivoire, Côte d’Ivoire; 6 Programme National de la Lutte Contre la Schistosomiase, les Geohelminthiases et la Filariose Lymphatique, Abidjan, Côte d’Ivoire; 7 Liverpool School of Tropical Medicine, Pembroke Place, Liverpool, United Kingdom; 8 Infectious Diseases Division, Department of Medicine, Washington University School of Medicine, St. Louis, Missouri, United States of America; 9 Veterans Affairs Research Service, Cleveland Veterans Affairs Medical Center, United States of America; 10 Fred and Pamela Buffett Cancer Center, University of Nebraska Medical Center, Omaha, NE, United States of America; Swiss Tropical and Public Health Institute, SWITZERLAND

## Abstract

**Background:**

A single co-administered dose of ivermectin (IVM) plus diethylcarbamazine (DEC) plus albendazole (ALB), or triple-drug therapy, was recently found to be more effective for clearing microfilariae (Mf) than standard DEC plus ALB currently used for mass drug administration programs for lymphatic filariasis (LF) outside of sub-Saharan Africa. Triple-drug therapy has not been previously tested in LF-uninfected individuals from Africa. This study evaluated the pharmacokinetics (PK), safety, and efficacy of triple-drug therapy in people with and without *Wuchereria bancrofti* infection in West Africa.

**Methods:**

In this open-label cohort study, treatment-naïve microfilaremic (>50 mf/mL, n = 32) and uninfected (circulating filarial antigen negative, n = 24) adults residing in Agboville district, Côte d’Ivoire, were treated with a single dose of IVM plus DEC plus ALB, and evaluated for adverse events (AEs) until 7 days post treatment. Drug levels were assessed by liquid chromatography and mass spectrometry. Persons responsible for assessing AEs were blinded to participants’ infection status.

**Findings:**

There was no difference in AUC_0-inf_ or C_max_ between LF-infected and uninfected participants (P>0.05 for all comparisons). All subjects experienced mild AEs; 28% and 25% of infected and uninfected participants experienced grade 2 AEs, respectively. There were no severe or serious adverse events. Only fever (16 of 32 versus 4 of 24, P<0.001) and scrotal pain/swelling in males (6 of 20 versus 0 of 12, P = 0.025) were more frequent in infected than uninfected participants. All LF positive participants were amicrofilaremic at 7 days post-treatment and 27 of 31 (87%) remained amicrofilaremic 12 months after treatment.

**Conclusions:**

Moderate to heavy *W*. *bancrofti* infection did not affect PK parameters for IVM, DEC or ALB following a single co-administered dose of these drugs compared to uninfected individuals. The drugs were well tolerated. This study confirmed the efficacy of the triple-drug therapy for clearing *W*. *bancrofti* Mf and has added important information to support the use of this regimen in LF elimination programs in areas of Africa without co-endemic onchocerciasis or loiasis.

**Trial registration:**

ClinicalTrials.gov NCT02845713.

## Author summary

Lymphatic filariasis is a mosquito-borne infection that causes disability in the form of lymphedema, hydroceles, and elephantiasis. It has been targeted for global elimination based on mass drug administration in the total population at risk including many people uninfected with LF. Recently, a single co-administered dose of IVM + DEC + ALB has been shown to be much more effective than the standard treatment with DEC + ALB for sustained clearance of Mf for 3 years based on studies in Papua New Guinea. This study confirms the efficacy and safety of triple-drug therapy for clearing of *Wuchereria bancrofti* Mf in an African population. The presence of LF did not affect drug levels and the medicines were well tolerated, with 28% and 25% rate of moderate AEs in infected and uninfected individuals respectively, and no severe or serious AEs, supporting the use of triple-drug therapy for mass drug administration. This study shows for the first time that triple-drug therapy also has a potent macrofilaricidal effect, as determined by the reduction in circulating filarial antigen and inactivation of worm nests one year following treatment.

## Introduction

Lymphatic filariasis (LF) is a mosquito-borne parasitic disease caused by nematode parasites. Host responses to the adult worms in lymphatic vessels cause stigmatizing morbidity (lymphedema, hydrocele, and elephantiasis) that can lead to chronic disability. Parasites that cause LF (*Wuchereria bancrofti*, and less commonly *Brugia malayi* and *Brugia timori*) are currently estimated to infect 80 million people in 52 tropical countries, with about 850 million at risk [1]. The World Health Organization (WHO) has targeted LF for global elimination by 2020 [2, 3]. The elimination effort is based on a mass drug administration (MDA) approach that uses one of three anti-filarial drug regimens; i) ivermectin (IVM) plus albendazole (ALB) in regions of Africa where *Onchocerca volvulus* is co-endemic, ii) ALB alone in areas where LF is co-endemic with *Loa loa*, and iii) diethylcarbamazine (DEC) plus ALB in LF endemic areas outside of Africa and in regions of Africa where *L*. *loa* and *O*. *volvulus* are not present [4]. Often, five or more annual rounds of MDA are required to reduce the community microfilarial reservoir to a level that cannot support sustained transmission of new infections by mosquitoes. Recently, a one time co-administered dose of IVM plus DEC plus ALB, or triple-drug therapy, was shown to achieve sustained microfilariae (Mf) clearance for three years in 96% of individuals with moderate to heavy LF infections in Papua New Guinea (PNG), compared to a lower cumulative clearance of Mf with standard therapy of DEC plus ALB administered once a year over the same period of time [5, 6]. Although the frequency of mild adverse events (AEs) was higher in the triple-drug regimen compared to the standard treatment of DEC plus ALB (27% versus 5%) [5], there were no serious AEs. Finally, it is unknown how LF infection itself might affect the pharmacokinetics (PK) and pharmacodynamics of these drugs. For example, inflammatory responses to chronic infections and the killing of Mf could affect the P450-mediated metabolism and PK of ALB in its first-pass conversion to ALB sulfoxide, the active metabolite [7]. This study evaluated the PK, safety, and efficacy of triple-drug therapy in men and women with and without *Wuchereria bancrofti* infection.

## Methods

### Study design and ethical review

This was an open-label cohort study of treatment naïve *Wuchereria bancrofti*-infected (n = 32) and uninfected (n = 24) adults residing in the Agboville district of Côte d’Ivoire, which is endemic for LF and non-endemic for onchocerciasis. The primary outcomes were drug levels and safety. The secondary outcomes were reduction in circulating Mf and parasite antigen levels, and inactivation of adult worm nests. Individuals who assessed adverse events and measured drug levels were blinded to LF infection status. The study protocol and related documents were approved by institutional review boards in Cleveland, USA (University Hospitals Cleveland Medical Center IRB #03-16-09) and in Côte d’Ivoire (Comité National d’Ethique et de la Recherche, CNER, N/Ref:022/MSLS/CNER-kp). This trial is registered at Clinicaltrials.gov (NCT02845713).

### Sources of medications and treatment

DEC (Banocide GlaxoSmithKline) was purchased for the study. ALB (GlaxoSmithKline) and IVM (Merck & Co. Inc.) were obtained from Ministry of Health stocks in Côte d’Ivoire used for the LF MDA program. A fixed dose of 400 mg ALB was used for all participants. IVM and DEC doses were 200 μg/kg and 6 mg/kg, respectively.

### Parasitology testing

Individuals were prescreened in their home villages for *W*. *bancrofti* infection with a rapid diagnostic test that detects circulating filarial antigenemia (the Alere Filariasis Test Strip, FTS, Alere, Inc, Waltham, MA, USA) [8]. Persons with positive FTS results had blood collected between 21:30 and 23:00 for Mf testing by membrane filtration with 1 mL of anticoagulated venous blood (5μM, Nuclepore Corp., Pleasanton, CA, USA). Two microscopists independently read Giemsa-stained filters to assess Mf load.

### Participants and enrollment

The study took place at the Centre de Recherche de Filariose Lymphatique d’Agboville, located at General hospital of Agboville, Côte d’Ivoire. Eligible participants included adults 18–70 years, with no acute illness, and no treatment with ALB or IVM within the past two years. Infected participants required >50 Mf/mL. Participants were considered uninfected if FTS strip was negative in whole blood and confirmed with plasma. Exclusion criteria included a positive pregnancy test; a history of chronic kidney or liver disease; a serum alanine transaminase, aspartate transaminase, or creatinine level >1.5 times the upper limits of normal; or blood hemoglobin <7 gm/dL. Biochemical tests were performed with a Piccolo biochemistry machine (Abbott Labs, Lake Bluff, IL, USA) and hemoglobin with a Hemocue Hb 201+ machine (HemoCue America, Brea, CA, USA). Individuals were also excluded if they had taken medications that could interfere with test drug metabolism within one week of study onset or if they had evidence of urinary tract infection on a spun urine sample (>10 neutrophils per high powered field by microscopy, 400x) or 3+ nitrate on dipstick (Diastix, Bayer Inc.). Because DEC can cause serious AEs in people infected with onchocerciasis [9], all individuals were tested by microscopic examination of skin snips taken from both iliac crests and by the presence of antibodies to a recombinant *O*. *volvulus* antigen (Ov16 Rapid Test, Standard Diagnostics Inc. Youngin, South Korea) [10]. Persons with microfiladermia or a positive Ov16 antibody test (n = 3) were excluded. All participants signed a written consent prior to screening and enrollment into the study.

### Blood collection and assessment of adverse events

Enrollment began on April 17, 2015 and continued through June 4, 2015. Groups of five participants of the same sex were brought to the research center the night prior to treatment for screening with baseline laboratory tests. For men,ultrasound examinations were performed. Investigators evaluating AEs and performing ultrasound examinations and laboratory tests were blinded to participants’ infection status. Participants remained at the research center until 72 hours post-treatment, then returned to their village for passive follow up until repeat examinations on day 7.

The first treatment dose of triple-drug therapy was given on April 18, 2015. Starting at 7 a.m., and within about 30 minutes after eating a typical Ivorian breakfast of wheat bread and eggs, all participants were treated with a single co-administered dose of triple-drug therapy. Venous blood samples were collected at 1, 2, 3, 4, 6, 8, 12, 24, 36, 48, 72 hours and 7 days after treatment, with aliquots of plasma stored at -20°C for later testing of drug levels. A peripheral intravenous catheter was placed for the first 12 hours due to frequent blood draws. Additional venous blood was collected between 21:30 and 23:00 at 39 hours, 7 days and 1 year for Mf testing with two 1 mL samples by membrane filtration. Biochemistry and urine tests were repeated at 24 and 48 hours and 7 days post-treatment.

After treatment, participants were monitored for AEs every 6 hours for the first 48 hours, then every 12 hours until 72 hours, and again at day 7 post-treatment. Passive surveillance for potential AEs was conducted by trained community health workers located in the participants’ home villages on days 4–6. New or worsening symptoms, changes in vital signs, or new abnormal findings on physical examination were considered to be treatment emergent adverse events (TEAEs) and were scored using a modified version of the National Cancer Institute Common Terminology Criteria for Adverse Events, v4.0. Blood pressure and heart rate were taken with an Omron-7 automatic blood pressure cuff with the participant in a seated position. Auricular temperatures were obtained using a digital thermometer.

Scrotal ultrasound examinations were performed on men in the supine position using a SonoScape S8 portable ultrasound system equipped with a 5–7.5Hz liner array transducer (International Diagnostic Devices, Inc, Las Vegas, NE, USA). Color and pulsed wave Doppler modes were used to differentiate lymphatic vessels from blood vessels. Adult worm nests were identified based on the characteristic bidirectional movement of the worms (the “filarial dance sign”) [11]. Abnormal ultrasound findings were digitally recorded. The presence or absence of worm nests was recorded, along with the number and size of worm nests, lymphatic dilatation, and hydroceles. Ultrasounds were repeated at 24, 48, 72 hours and 7 days and 1 year after treatment.

### Pharmacokinetic methods

#### Chemicals and materials

Pharmaceutical grade DEC, IVM, ALB, albendazole sulfoxide (ALB-OX), albendazole sulfone (ALB-ON), oxibendazole (OBZ), and deuterated diethylcarbamazine (D3-DEC) were obtained from Sigma-Aldrich, St Louis, MO, USA. Ultrapure water was obtained from a Barnstead Ultrapure Thermo-Scientific water purification system. Methanol, tetrahydrofuran, acetic acid, formic acid, and acetonitrile were purchased from Fisher Scientific (Fair Lawn, NJ, USA). Centrifuge tube filters were obtained from Corning Co. (Corning, NY, USA). Agilent (Santa Clara, CA, USA) bond Elute C18 solid phase extraction cartridges (50mg/mL) were used to extract drugs from plasma. All other chemical reagents were from Sigma (St. Louis, MO, USA) and were of analytical grade.

#### Liquid chromatographic and mass spectrometric conditions

Plasma concentrations of DEC, ALB, ALB-OX, and ALB-ON were determined using a validated liquid chromatography-mass spectrometric (LC-MS/MS) method [12]. For IVM, plasma concentrations were determined using high performance liquid chromatography (HPLC) with fluorescence detection as previously described [13].

Calibration standards (CS) were prepared by spiking 10 μL of mixed working standard solution into 100 μL of human plasma to obtain a concentration range of 1–2000 ng/mL for DEC, 0.5–1000 ng/mL for ALB-OX, and 0.1–200 ng/mL for ALB and ALB-ON. Quality control samples (QCs) were prepared at four different concentrations: 1, 5, 500 and 1500 ng/mL for DEC; 0.5, 2, 200 and 750 ng/mL for ALB-OX; and 0.1, 0.5, 40 and 150 ng/mL for both ALB and ALB-ON. The lower limit of quantification (LLOQ), low quality control (LQC), middle quality control (MQC), and high-quality control (HQC) were prepared separately in five replicates, independent of the CS. A 10 μL spiking of internal standard from a mixed internal stock of OBZ and D3-DEC solution was added to all CS and QCs. The linear range of the calibration curve for IVM was 0.20–400 ng/mL from 0.20 mL plasma.

### Statistical analysis

Microfilarial counts were expressed as Mf/mL and log transformed after adding 1, and geometric mean values (GM) were used as measures of central tendency to normalize the results. Baseline sample characteristics between individuals infected or uninfected with LF and the impact of treatment of Mf and worm nests were compared using the chi-squared test or the Mann-Whitney test using GraphPad Prism, version 6. For the analysis of the impact of treatment on the inactivation of worm nests, only men who had detectable worm nests at baseline were included. For the sample size calculation we used methods as previously described [14]. We wanted to observe less than 50% difference in clearance of drugs between LF-infected and uninfected individuals. Considering a variance of ALB-OX (the most variable of the drugs [6]) of approximately 50% from the mean, with an alpha = 0.05 and power = 0.8, the number of participants required would be 20–24 per study population. The PK parameters were determined by noncompartmental analysis using Phoenix WinNonlin version 6.3 (Pharsight Corporation, CA, USA). For analytes that did not have at least eight calculated values, the mean and standard deviation were not calculated. Where R^2^adj was <0.85, this parameter was reported as NE (not estimated). Total drug exposure up to the last measured concentration (AUC_0-last_) was calculated using the linear trapezoidal method for ascending concentrations and the logarithmic trapezoidal method for descending concentrations. The AUC_0-last_ was defined as the area under the concentration-time curve from the time of dose until the last concentration above lower limit of quantitation. Area under the concentration-time curve from 0 to infinity (AUC_0-inf_) was calculated using the formula AUC_0-t_ + C(last) / K_el_, where C(last) is the last quantifiable concentration. Half-life (t_1/2_) was calculated using the formula ln(2)/ K_el_. Maximum concentration (C_max_) and time to reach maximum concentration (T_max_) were taken directly from the observed data. Apparent volume of distribution (V_z_/F) was calculated as dose/(K_el_ *AUC_0-inf_) and apparent clearance (CL/F) calculated as dose/AUC_0-inf_, according to standard procedures.

PK estimate comparisons between uninfected and infected participants were performed using the Kruskal-Wallis test using the JMP software version 13 (Cary, NC, USA).

## Results

### Study population

**Fig 1:**
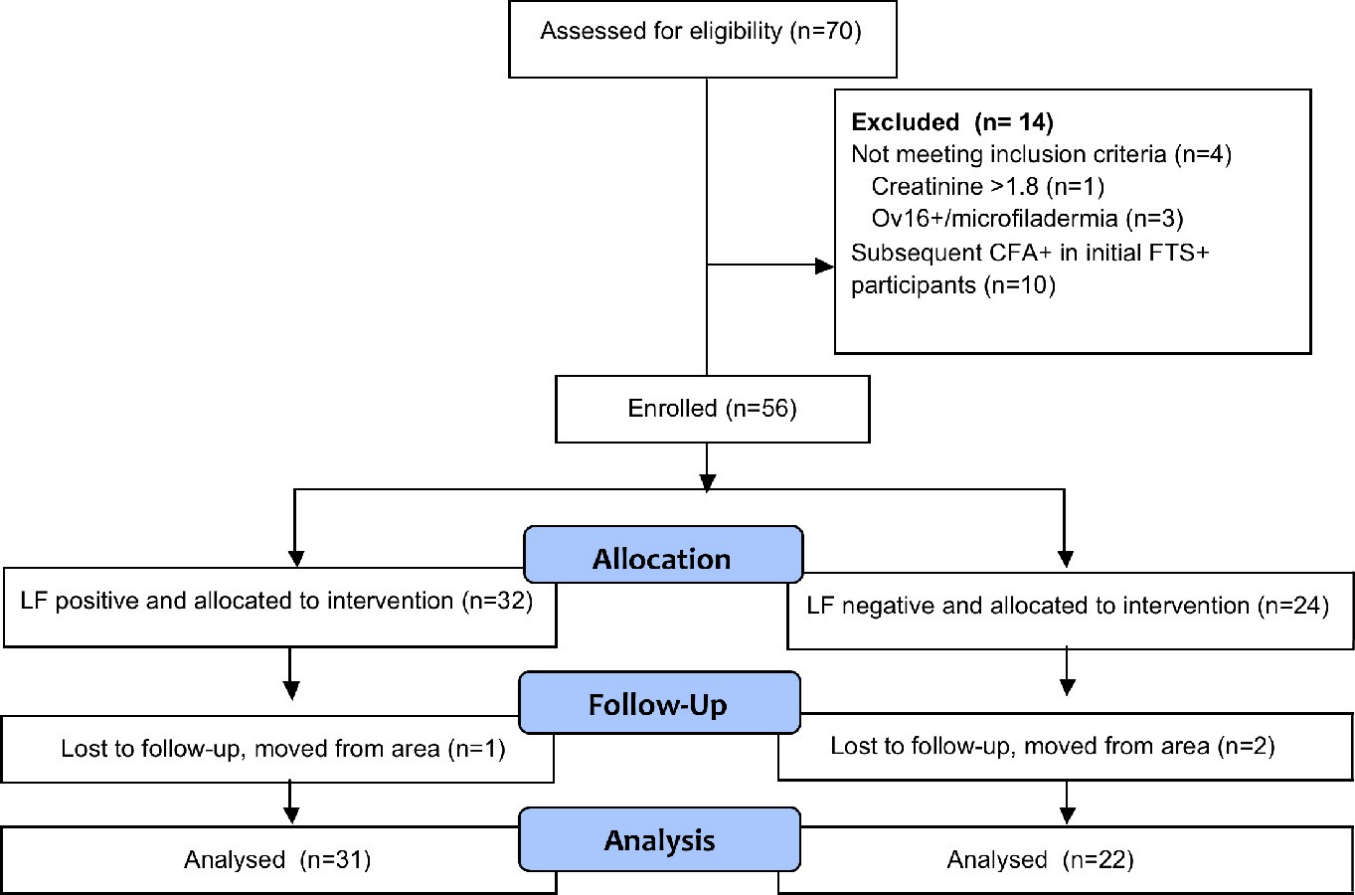
Screening, enrollment, PK study, and one-year follow-up to assess drug efficacy.

Screening of 1,534 adults yielded 70 eligible individuals, with 36 LF-infected (FTS+, Mf+) and 34 uninfected (FTS-, Mf-). These individuals then underwent full screening (Fig 1). Three participants were excluded due to evidence of onchocerciasis infection and one individual was excluded because of elevated creatinine. Excluded participants were treated with IVM + ALB and returned to their village. Ten participants initially treated in the LF-uninfected (FTS-) group were subsequently found to be LF-infected based on repeated examination of plasma with FTS assay and excluded from subsequent analysis per protocol. Baseline demographics were similar in the two groups (Table 1). Thirty-two (57%) of all participants were men. One year following treatment, 31 of 32 (97%) infected and 22 of 24 (92%) uninfected participants were re-examined for the presence of LF infection (FTS, Mf, and ultrasound in males). The three individuals not examined had all moved from the area. Two uninfected men seen at follow-up did not have a repeat ultrasound.

**Table 1:** Study population.

Characteristics	Infected	Uninfected
	(n = 32)	(n = 24)
Age, median (IQR) ^†^, years	44.1	36.2
	(37,54)	(27,43)
Male sex (%)	20 (63)	12 (50)
Microfilaria count, geometric mean,	146.3	0
Mf/mL (range)	(51–739)	
Mean FTS score	2.35	0
No. FTS +1 (% of infected)	2 (6)	0
No. FTS +2 (% of infected)	16 (50)	0
No. FTS +3 (% of infected)	14 (44)	0
Men with worm nests divided by the number examined (%)	14/20 (77)	0

^†^Interquartile range

### Pharmacokinetic data

**Fig 2:**
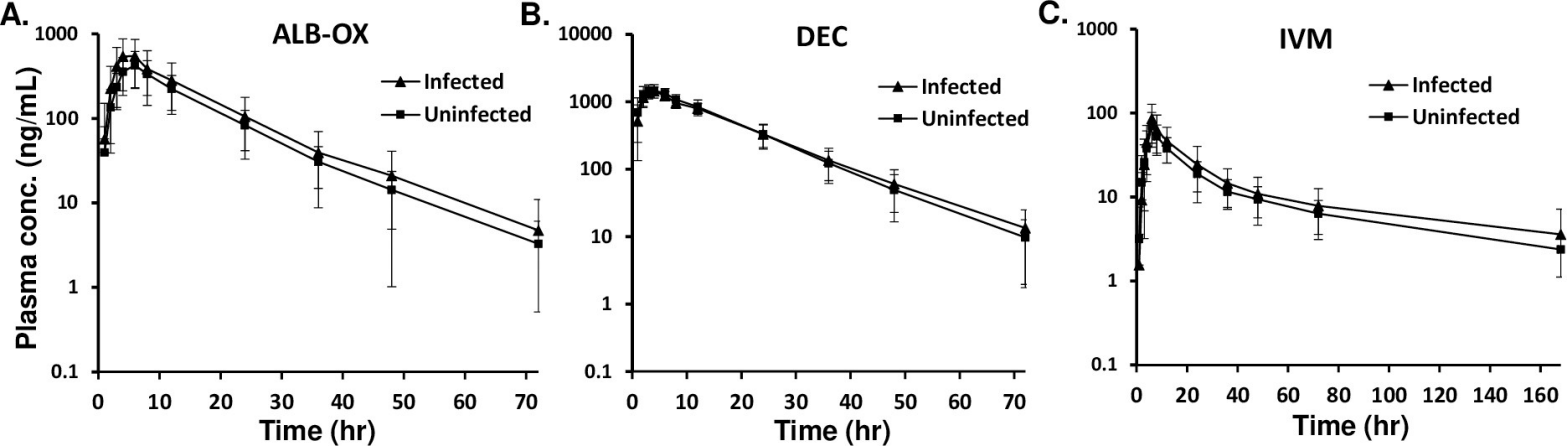
Plasma concentration-time profiles of (**A**) ALB-OX, (**B**) DEC, and (**C**) IVM, after a single dose of IVM+DEC+ALB stratified by LF infection status (infected = 32, uninfected = 24). Mean (±SD) are shown.

**Fig 3:**
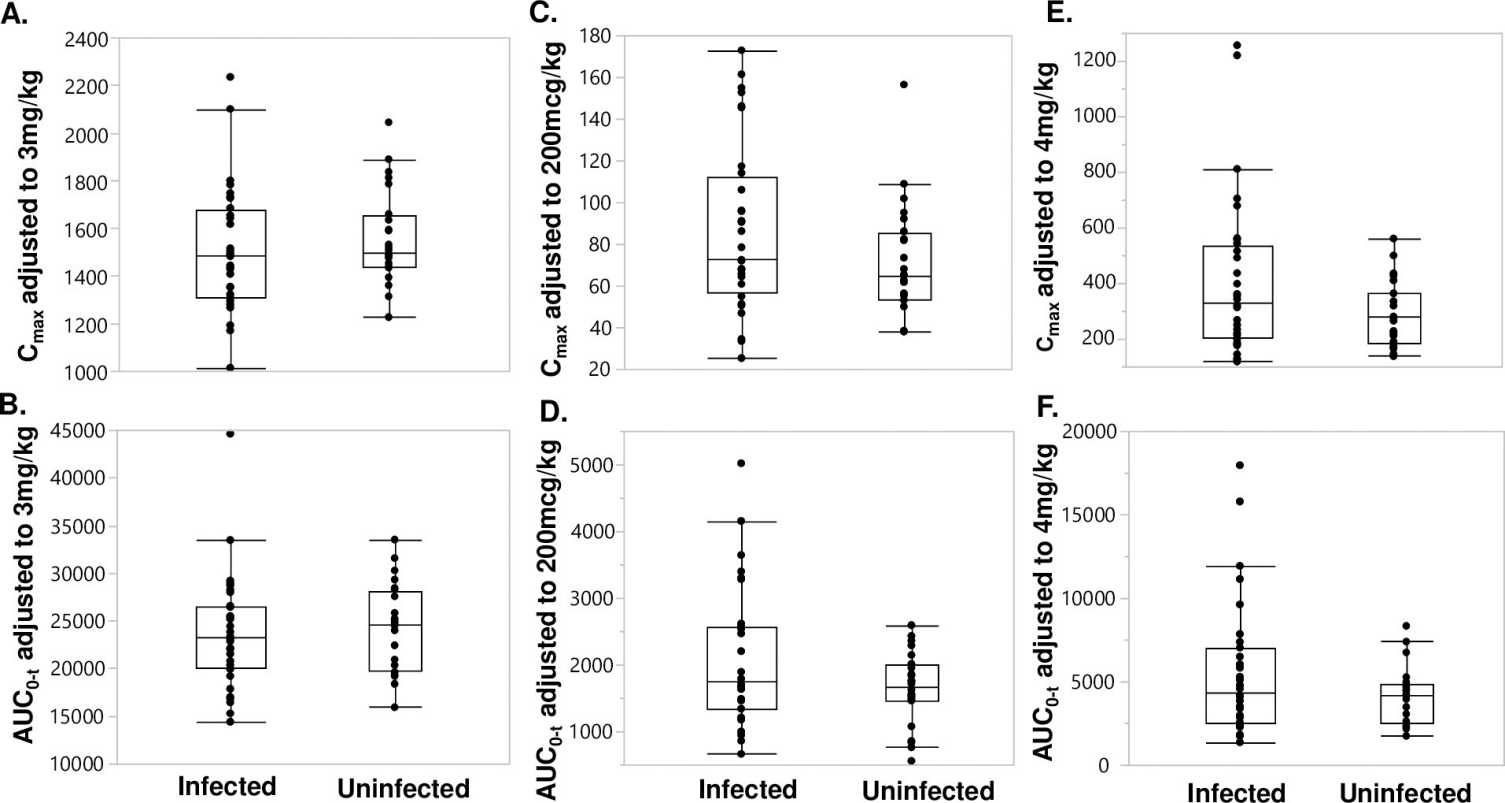
Distribution of dose adjusted C_max_ and AUC_0-t_ of DEC (**A** and **B**), IVM (**C** and **D**), and ALB-OX (**E** and **F**) stratified by LF infection status. The median, 25^th^, and 75^th^ quartiles and 95% CI are shown. Significance was assessed with the Kruskal-Wallis test and all P values were >0.05. There were 32 LF-infected and 24 uninfected participants.

**Fig 4:**
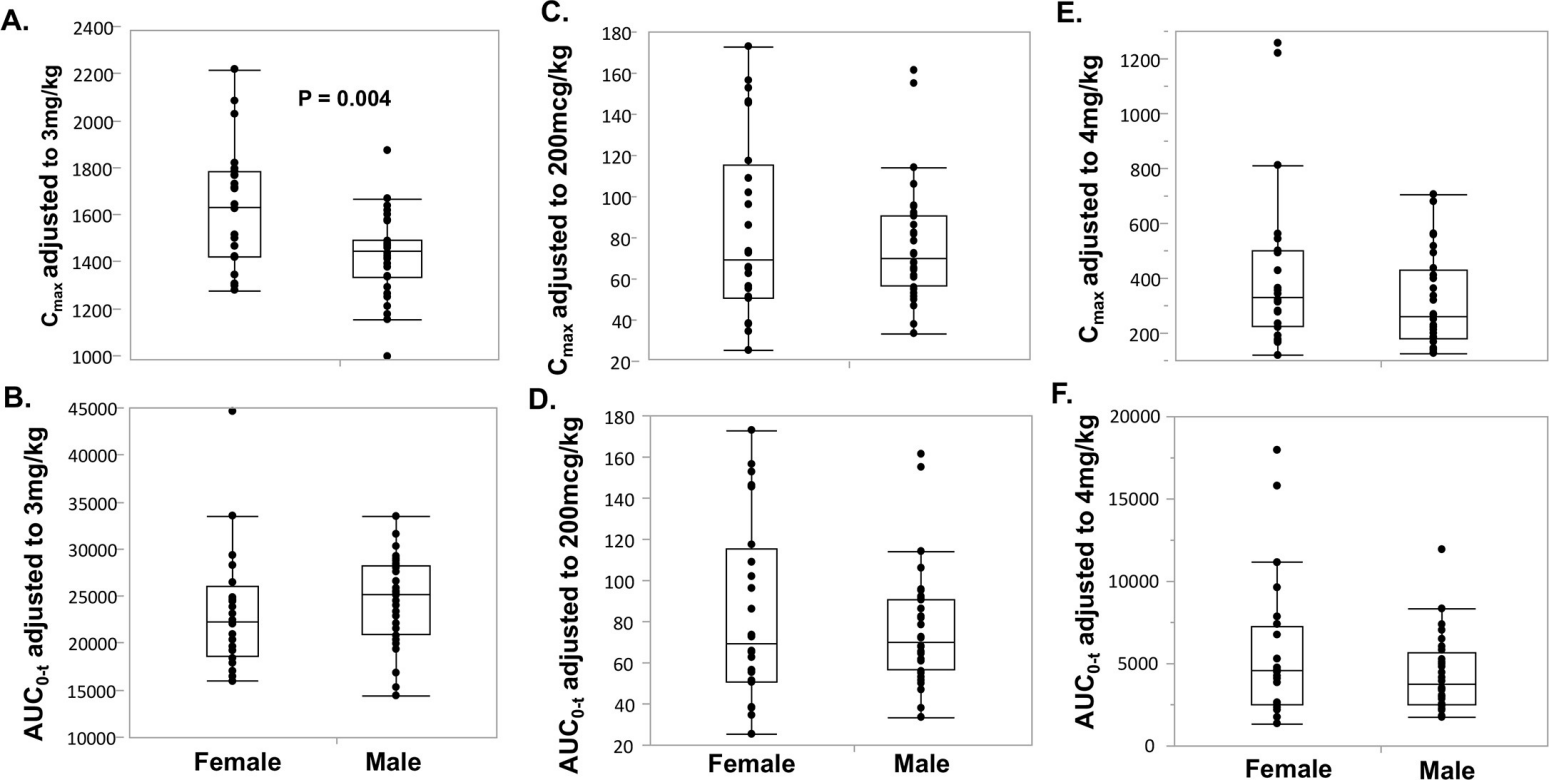
Distribution of dose adjusted C_max_ and AUC_0-t_ of DEC (**A** and **B**), IVM (**C** and **D**), and ALB-OX (**E** and **F**) stratified by sex. The median, 25^th^, and 75^th^ quartiles and 95% CI are shown. Significance was determined by using the Kruskal-Wallis test. P values for all comparison were >0.05 except C_max_ for DEC (P = 0.004). There were 24 women and 32 men.

The mean plasma concentration-time profiles of ALB-OX (the active metabolite of ALB), DEC, and IVM are shown in Fig 2, and of ALB and ALB-ON are shown in S1 Fig. There was no difference in plasma concentration for all the drugs between infected and uninfected individuals for all time points examined. Maximum concentrations of ALB-OX were substantially higher than corresponding concentrations for ALB, but showed less variability compared to ALB and ALB-ON, a secondary metabolite. The main PK parameters (median and range) of ALB, ALB-OX, ALB-ON, DEC, and IVM for all 56 subjects are shown in S1 Table. The main PK parameters (median and range) stratified by infection status or by sex are shown in S2 and S3 Tables. Overall, the DEC T_max_ occurred at a median time of 4.0 hours post-treatment, with a reported C_max_ of 1,522 ng/mL. The median t_1/2_ for DEC was 9.5 hours. The CL/F and V_z_/F of DEC were 8.1 L/hour and 111 L, respectively. The median C_max_ for DEC was not different based on infection status (Fig 3A); however, C_max_ was higher in female compared to male participants (P< 0.05, Fig 4A). The AUC_0-t_ for DEC with LF and without LF (Fig 3B), or based on sex (Fig 4B) was not different (P>0.05). The median T_max_ for IVM was 6.0 hours, with a median t_1/2_ of 48.1 hours (S1 Table). The CL/F and V_z_/F of IVM were 6.8 L/hour and 466.7 L, respectively. IVM C_max_ and AUC_0-t_ was not significantly different (P>0.05) when comparing participants with and without LF (Figs 3C and 3D), as well as treatment group comparisons based on sex (Fig 4C and 4D). The median T_max_ for ALB-OX was 5.0 hours, with a median t_1/2_ of 8.9 hours (S1 Table). The CL/F and V_z_/F of IVM wer2 L/hour and 848 L, respectively. For ALB-OX, the C_max_ and AUC_0-t_ parameters were not significantly different (P>0.05) in the presence or absence of LF (Fig 3E and 3F) and for sex basis (Fig 4E and 4F).

### Safety and tolerability

Since mild subjective complaints were common at baseline, new subjective findings (e.g. symptoms) and objective findings (e.g. fever, presence of hematuria, hemodynamic changes), or worsening after treatment of objective and/or subjective observations compared to baseline, were considered to be study-related AEs. Every participant had at least one AE (Table 2). Headache, abdominal pain, and muscle/joint pain were the most common symptoms, followed by diarrhea and fatigue. There was no difference in the frequency of subjective AEs between LF-infected and uninfected individuals. Women were more likely to have multiple subjective AEs (21 (88%) versus 22 (69%) for men, P = 0.02), but there was no difference in the severity of AEs between sexes. For all participants, AEs were most common between 18 and 48 hours post-treatment. With respect to objective AEs, 16 of 32 (50%) LF-infected subjects had fevers versus 4 of 24 (17%) uninfected participants post-treatment (P = 0.01). Individuals with fevers were not treated with antipyretics to allow for evaluation of the kinetics of post-treatment fever. Fevers occurred most commonly between 18 and 42 hours. Frequency and severity peaked at 36 hours in LF-infected subjects. All fevers resolved by 72 hours after treatment. Scrotal swelling and pain occurred in 6 of the 20 (30%) LF- infected men, but this was not observed in uninfected men (P = 0.04). The onset of scrotal swelling and pain occurred from 48 to 96 hours post-treatment. Hematuria (based on urine dipstick, but not by microscopy) was detected in 10 of 32 (31%) LF- infected individuals, compared to 3 of 24 (13%) uninfected participants (P = 0.1, Table 2). Hematuria was predominantly seen at 24 and 48 hours and was resolved by day 7.

**Table 2:** Adverse events (AEs) following treatment with a single dose of IVM, DEC, and ALB.

	Number of participants with AEs (percent)	
	Infected	Uninfected
	(n = 32)	(n = 24)
At least one AE	32 (100)	24 (100)
Individuals with two or more AEs	30 (94)	20 (88)
Severe or Serious AEs	0	0
Fever ≥ 37.5°C^§^	16 (50)	4 (17)
Hemodynamic changes^¶^	7 (22)	6 (25)
Hematuria	10 (31)	3 (13)
Overall Grade 1 AEs (subjective)	32 (100)	23 (96)
Overall Grade 2 AEs (subjective)	9 (28)	6 (25)
Subjective AEs by symptoms		
Headache	15 (47)	11 (46)
Abdominal Pain	14 (44)	11 (46)
Diarrhea	14 (44)	7 (29)
Fatigue	10 (31)	6 (25)
Muscle/Joint ache	10 (31)	4 (17)
Lightheaded	3 (9)	7 (29)
Scrotal pain/swelling*	6/20 (30)	0
Itching	4 (13)	1 (4)
Cough	2 (6)	3 (13)
Swelling	3 (9)	1 (4)
Back pain	1 (3)	3 (13)
Rash	1 (3)	2 (8)
Nausea/vomiting	0	1 (4)
Other^†^	4 (13)	0

^§^Temperature increased ≥0.9°C post-treatment relative to pre-treatment to at least 37.5°C based on auricular temperature

^¶^Systolic blood pressure decrease of 20mmHg and <100 or diastolic blood pressure decrease of 10mmHg and <50, P = 0.278

*P = 0.025

^†^one person each with eye selling, lymph node swelling, insomnia and paresthesia

### Efficacy

**Fig 5:**
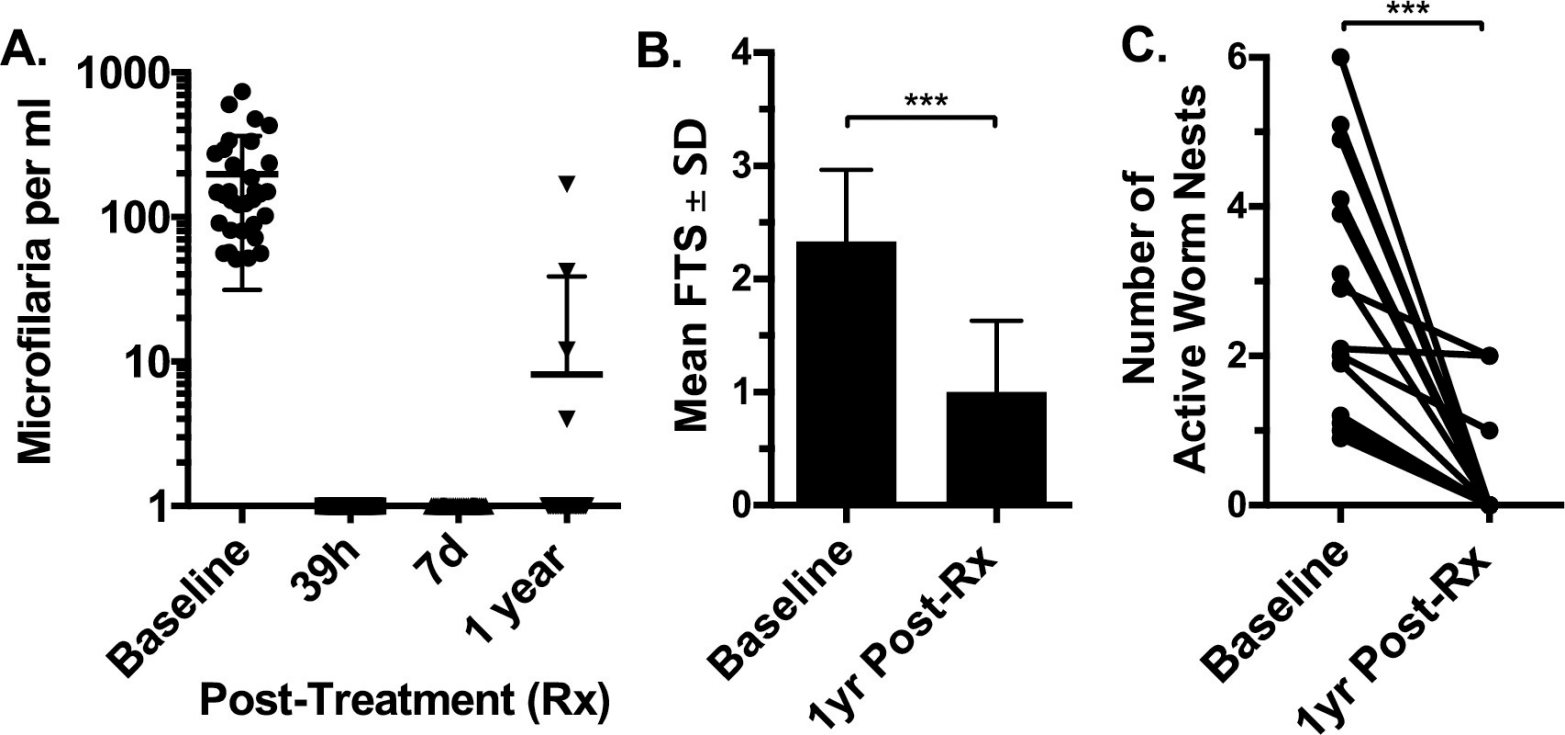
The efficacy of a single co-administered dose of the triple-drug regimen before and after treatment with respect to (**A**) Mf counts, (**B)** circulating filarial antigen levels as determined by mean filarial test strip (FTS) scores at 1 year after treatment, and (**C**) the number of active worm nests at one-year follow-up. Significance was determined by using the Mann Whitney test for FTS and worm nests (*** <0.001).

Pre-treatment infection parameters are shown in Table 1. Infected men and women had very similar Mf counts and circulating filarial antigen test scores (geomean = 141 Mf/mL and average FTS of 2.3, and geomean = 141.82 Mf/mL and mean FTS of 2.4, respectively). At 39 hours and 7 days post-treatment, all LF-infected participants were Mf negative, and 27 of 31 (87%) were Mf negative 1 year after treatment (Fig 5A). FTS scores decreased significantly after treatment (Fig 5B, P<0.001), and 6 of 31 (19%) participants were FTS negative. All 32 men enrolled in the study had scrotal ultrasound at baseline. Adult worm nests were detected in 14 of 20 (70%) infected men and in 0 of 12 uninfected men. The mean number of worm nests at baseline in positive men was 2.6 (range 1–6). The mean maximum diameter of worm nests was 3.8 mm (range 1.4–7.6 mm). Ultrasounds were repeated on all men at 24, 48, 72 hours and 7 days following treatment. No significant changes were seen in the number or size of worm nests, the degree of lymphatic dilatation or the development of hydroceles at those time points. Ultrasound examinations were performed for 30 men at 1 year after treatment (20 of 20 infected men and 10 of 12 uninfected men). No new worm nests were seen in men of either group. Of the 14 men who had worm nests at baseline, 11 had no detectable worm nests at one year (79% reduction, p<0.001, Fig 5C). Of the three men who had worm nests visible at 1 year, two had a reduced number relative to baseline, and one had no change. One of the men who still had detectable worm nests, though decreased, also had detectable Mf. The other two men were amicrofilaremic at 1 year.

### Lack of relationship between drug levels and sustained clearance of Mf

**Fig 6:**
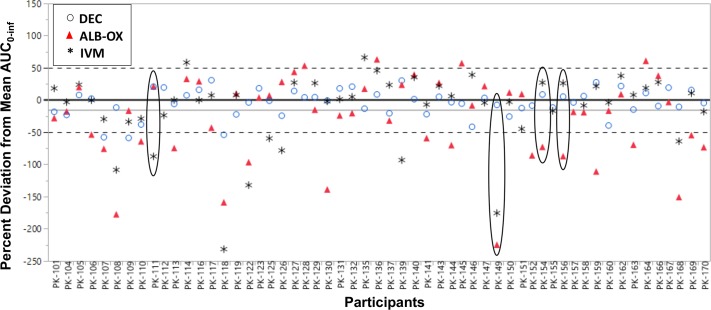
Variation in drug levels from the mean AUC_0-inf_ for all 56 study participants. DEC is depicted as blue circles, ALB-OX as red triangles, and IVM as black asterisks. Four Mf+ individuals at baseline failed to completely clear Mf at 1 year following triple- drug therapy and are highlighted (ellipses).

Four individuals failed to have sustained clearance of Mf at 1 year following triple-drug treatment (Fig 5). To determine whether incomplete Mf clearance might result from reduced drug levels compared to levels in individuals with sustained clearance, we calculated the variance of all three drugs from the mean AUC_0-inf_ for each participant (Fig 6). Although there was considerable variability in drug levels among individuals, persons who were microfilaremic at 1 year (circled) had similar overall drug levels to those observed in participants with sustained Mf clearance.

## Discussion

Results from this study show that the presence of moderate to heavy LF infection does not affect IVM, DEC or ALB drug levels or their PK parameters following a single co-administered dose of the triple-drug regimen. Triple-drug therapy was well tolerated in both LF-infected and uninfected individuals and was effective for clearing Mf of *W*. *bancrofti* in Ivorian participants for up to 1 year after treatment. These studies provide important additional information in support of the use of triple-drug therapy for MDA in LF endemic areas where onchocerciasis and loaisis are not present.

LF infection did not affect the PK and pharmacodynamics (PD) of IVM, DEC or ALB, based on the observation that the kinetics of drug levels and derived PK parameters for all three drugs were the same irrespective of whether an individual was LF-infected or not. There was considerable variability in plasma ALB and IVM drug levels among individuals. Both drugs, and especially ALB, have been shown to have highly variable gastrointestinal absorption [15], although this has not been shown to affect drug efficacy. By contrast, plasma levels of DEC showed relatively little variation among individuals. This is likely a consequence of good drug bioavailability of DEC. Drug levels did not differ between sexes with the exception of C_max_ for DEC, which was higher in women. Compared to ALB and IVM, which are lipophilic and thus have a large V_z_/F [16], DEC has a low V_z_/F that is more closely associated with the ideal body weight of an individual [17]. This suggests that high DEC concentrations might occur in persons who are overweight if they are dosed based on actual body weight with no maximum dose. However, future studies are needed to evaluate the relation with weight, DEC dosing, and systemic concentrations. Of note, previous studies have shown that the addition of IVM to DEC plus ALB or IVM to ALB does not significantly alter the PK parameters of individual drugs compared to those observed in various combinations, including the triple-drug combination [6, 18].

All participants complained of one or more AEs, which is comparable to that observed in PNG study participants who were treated with the triple-drug regimen (83%) in a similarly designed PK study [6]. The high rate of reported AEs in infected and uninfected participants is probably related to the high frequency of symptom assessment and to effects of confining normally active adults to a hospital ward for three days. One of the most common AEs was back pain, which was relieved by getting up and walking around. Another common AE was dyspepsia, which could possibly be attributed to changes from the normal village diet to that provided while in the study clinic. The two AEs that differed between infected and uninfected adults were fever and scrotal pain and swelling. Fever is a well-known side effect of LF treatment that is associated with the host inflammatory reaction to dying Mf [19, 20]. The inflammatory response is exacerbated in both prevalence and severity with higher blood Mf counts [6, 21, 22]. Scrotal pain is likely a reaction to dying adult worms. The ingestion of the drugs themselves have well-known side effects independent of their impact on helminth infections; for IVM this can include dizziness, joint pain, and skin irritation, and for DEC and ALB, common symptoms are nausea, abdominal discomfort, and headache.

Treatment with triple-drug therapy rapidly cleared Mf in all participants by 39 hours, and all but four individuals remained amicrofilaremic (87% clearance) at 1 year following treatment. This is comparable to, although somewhat less than, the sustained blood Mf clearance levels (96%) of LF-infected subjects in PNG 1 year post-treatment with triple-drug therapy, even though participants from PNG had 4.5 to 10 times higher Mf levels [6, 22]. Failure to sustain Mf clearance was not attributable to reduced drug levels (Fig 6). It is possible that some participants were re-infected during the follow-up period, although this is less likely in one year because the prepatent period for *W*. *bancrofti* is about 4 months [23] and we failed to observe any new worm nests in participants. Results from a larger clinical trial of triple-drug therapy in Côte d’Ivoire may shed further light on variability in responses to this regimen. Our results suggest that triple-drug therapy killed many adult worms, as evidenced by reduced circulating filarial antigen levels as assessed by lower FTS scores and the inactivation of worm nests observed by ultrasound after treatment. Inactivation of worm nests following treatment has been interpreted as evidence of a macrofilaricidal effect [24], and one study confirmed this by histological examination of surgically removed worm nests after DEC treatment [25]. Measurement of circulating filarial antigen levels provides a second means of assessing macrofilaricidal activity of antifilarial medications, and antigen levels are believed to correlate with adult filarial burdens [26].

Limitations of this study include the exclusion of children and people with chronic disease (who would normally be included in MDA programs) and the relatively small sample size. Community-wide safety trials including almost 26,000 people in five countries have now been completed. These showed that triple-drug therapy was well tolerated and suggested that it should be as safe as the two-drug MDA regimen DEC + ALB that has been widely used outside of sub-Saharan Africa. Based on these and other studies, WHO changed its policy to recommend triple-drug therapy for MDA in areas that are non-endemic for onchocerciasis or loiasis and unlikely to eliminate LF by the year 2020 [27].

## Supporting information

S1 Fig Plasma concentration-time profiles of (**A**) ALB and (**B**) ALB-ON after a single dose of IVM+DEC+ALB stratified by LF infection status (infected = 32, uninfected = 24). Mean (±SD) are shown.
(TIF)

S1 Table Non-compartmental PK estimates for ALB, ALB-OX, ALB-ON, DEC, and IVM in all adult participants (N = 56) after a single dose of ALB (400mg), DEC (6 mg/kg), and IVM (0.2mg/kg). Median values and range are shown, n = 56.
(DOCX)

S2 Table Non-compartmental PK estimates for ALB, ALB-OX, ALB-ON, DEC, and IVM in LF-uninfected (n = 24) and infected (n = 32) adult subjects after receiving a single combined dose of ALB (400mg), DEC (6 mg/kg), and IVM (0.2mg/kg). Median values and range are shown.
(DOCX)

S3 Table Non-compartmental PK estimates for ALB, ALB-OX, ALB-ON, DEC, and IVM stratified by sex, for men (N = 32) and women (n = 24) adult participants after receiving a single combined dose of ALB (400mg), DEC (6mg/kg), and IVM (0.2mg/kg), Median values and range are shown. (DOCX)
