# Supplementary figures and images for: Pharmacokinetics, safety, and efficacy of a single co-administered dose of diethylcarbamazine, albendazole and ivermectin in adults with and without Wuchereria bancrofti infection in Côte d’Ivoire
Source: PLoS Negl Trop Dis. 2019 May 20;13(5):e0007325. doi: 10.1371/journal.pntd.0007325 (PMC6550417; doi:10.1371/journal.pntd.0007325)

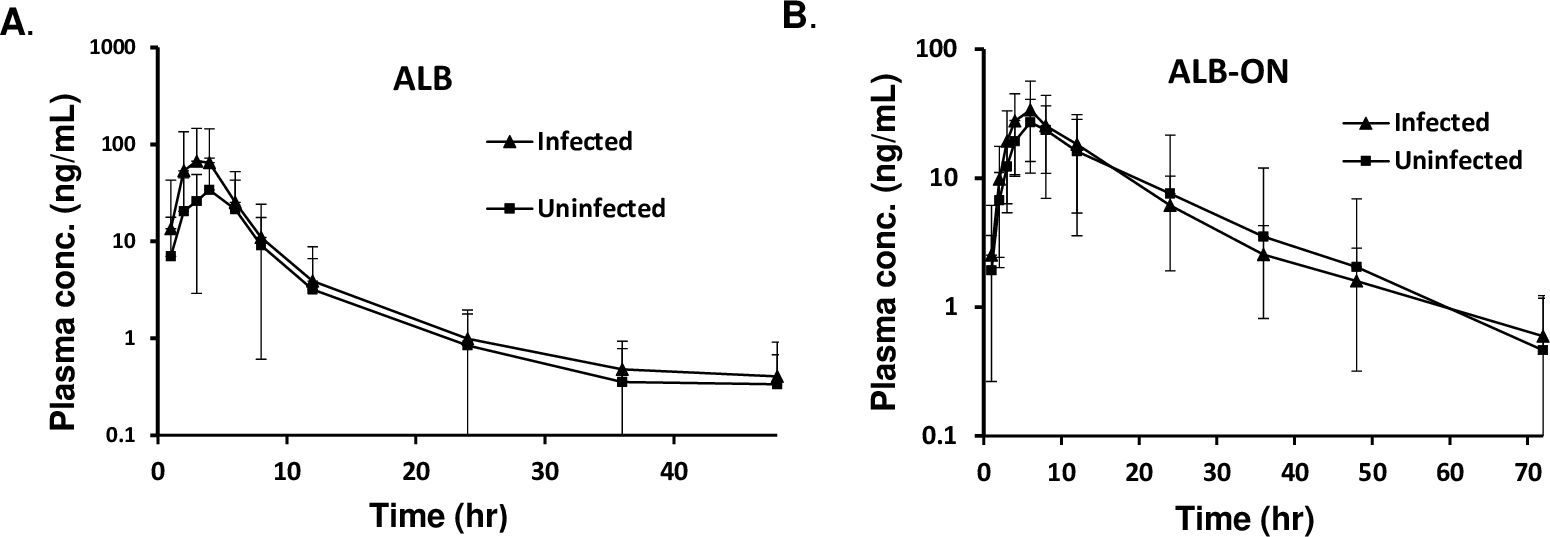

Supplement: S1 Fig — Plasma concentration-time profiles of (A) ALB and (B) ALB-ON after a single dose of IVM+DEC+ALB stratified by LF infection status (infected = 32, uninfected = 24). Mean (±SD) are shown. (TIF) [file pntd.0007325.s001.tif]
