# Supplementary material for: Pharmacokinetics, safety, and efficacy of a single co-administered dose of diethylcarbamazine, albendazole and ivermectin in adults with and without Wuchereria bancrofti infection in Côte d’Ivoire
Source: PLoS Negl Trop Dis. 2019 May 20;13(5):e0007325. doi: 10.1371/journal.pntd.0007325 (PMC6550417; doi:10.1371/journal.pntd.0007325)
Supplement: S1 Table — Median values and range are shown, n = 56. (DOCX) [file pntd.0007325.s002.docx]

**Supplementary Table S1:** Non-compartmental pharmacokinetic estimates for albendazole (ALB), albendazole sulfoxide (ALB-OX), albendazole sulfone (ALB-ON), diethylcarbamazine (DEC) and ivermectin (IVM) in all adults participants after a single dose of ALB (400mg), DEC (12mg/kg) and IVM (0.2mg/kg). Median values and range are shown.

| **PK Parameters** | **Median (Range)** | | | | |
| --- | --- | --- | --- | --- | --- |
|  | **ALB** | **ALB-OX** | **ALB-ON** | **DEC** | **IVM** |
| **C_max_ (ng/mL)** | 33.6  (2.7 - 336.0) | 454.7  (204.2 - 1398.2) | 27.2  (12.2 - 126.9) | 1522.8  (1073.6 - 2139.9) | 73.0  (25.2-179.1) |
| **T_max_ (hr)** | 3.5  (2.0 - 6.0) | 5.0  (3.0 - 12.0) | 6.0  (3.0 - 24.0) | 4.0  (1.0 - 12.0) | 6.0  (2.0-12.0) |
| **t_1/2_ (hr)** | 8.9  (1.4 - 22.4) | 9.2  (4.8 - 18.6) | 10.3  (5.0 - 23.4) | 9.5  (6.5 - 14.7) | 48.1  (19.5-98.9) |
| **AUC_0-t_ (hr*ng/mL)** | 176.1  (18.0 - 1461.0) | 6322.9  (2323.8 - 20029.9) | 355.7  (134.2 - 2679.4) | 23719  (15265.7 - 35088.1) | 1817.7  (557.5-5424.9) |
| **AUC_0-∞_(hr*ng/mL)** | 179.7  (18.2 - 1328.6) | 6454.7  (2331.1 - 20389.8) | 377.4  (137.6 - 2723.0) | 23807  (15305.4 - 35171.7) | 1969.3  (593.6-5824.5) |
| **V_z/F_ (L)** | 22874.5 (3450.9 -402663.8) | 847.5  (257.7 - 2672.0) | 15800.3  (1994.2 - 58789.8) | 111.0  (81.2 - 170.8) | 466.7  (122.1-1661.8) |
| **Cl/F (L/hr)** | 2226.5  (301.1 - 21951.7) | 62.0  (19.6 - 171.6) | 1059.9  (146.9 - 2907.3) | 8.1  (5.1 - 13.3) | 6.8  (2.1-20.2) |
| **C_max_ adjusted to dose (ng/mL)** | 22.4  (1.6 - 262.7) | 315.8  (118.4 - 1256.0) | 17.3  (7.1 - 114.2) | 1488.9  (1012.4 - 2233.4) | 70.0  (25.2-172.9) |
| **AUC_0-t_ adjusted to dose (hr*ng/mL)** | 109.0  (11.5 - 1194.2) | 4196.7  (1347.8 - 17957.6) | 231.3  (77.8 - 1580.9) | 23903.2  (14395.9 - 44627.9) | 1706.1  (557.5-5018.0) |
| **AUC_0-INF_ adjusted to dose (hr*ng/mL)** | 116.7  (11.7 - 1195.7) | 4377.5  (1352.0 - 18107.6) | 247.5  (79.8 - 1606.6) | 24060.1  (14433.4 - 45028.3) | 1863.8  (593.6-5387.7) |

C_max_ maximum observed plasma concentration after administration, T_max_ observed time to reach C_max_, CL/F elimination clearance, V_z/_/F apparent volume of distribution, t_1/2_ terminal elimination half-life, AUC_0-t_ area under the plasma concentration time curve after the last dose from zero time to last time point, AUC_0-∞_ predicted area under the plasma concentration time curve after the last dose from zero time to infinity.
