# Supplementary material for: Pharmacokinetics, safety, and efficacy of a single co-administered dose of diethylcarbamazine, albendazole and ivermectin in adults with and without Wuchereria bancrofti infection in Côte d’Ivoire
Source: PLoS Negl Trop Dis. 2019 May 20;13(5):e0007325. doi: 10.1371/journal.pntd.0007325 (PMC6550417; doi:10.1371/journal.pntd.0007325)
Supplement: S2 Table — Median values and range are shown. (DOCX) [file pntd.0007325.s003.docx]

**Supplementary Table S2.** Non-compartmental pharmacokinetic estimates for albendazole (ALB), albendazole sulfoxide (ALB-OX), albendazole sulfone (ALB-ON), diethylcarbamazine (DEC) and ivermectin (IVM) in uninfected (n=24) and infected with Lymphatic filariasis (LF) (n=32) adults subjects after receiving a single combined dose of ALB (400mg), DEC (12mg/kg) and IVM (0.2mg/kg). Median values and range are shown.

| **PK Parameters (Units)** | **Median (Range)** | | | | | | | | | |
| --- | --- | --- | --- | --- | --- | --- | --- | --- | --- | --- |
|  | **ALB** | | **ALB-OX** | | **ALB-ON** | | **DEC** | | **IVM** | |
|  | Uninfected | Infected | Uninfected | Infected | Uninfected | Infected | Uninfected | Infected | Uninfected | Infected |
| **C_max_ (ng/mL)** | 31.4  (3.1 - 173.1) | 39.6  (2.7 - 336) | 405.2  (211.7 - 857.3) | 556.1  (204.2 - 1398.2) | 25.8  (12.2 - 71.1) | 30.6  (12.2 - 126.9) | 1524.9 (1234.3 - 2139.9) | 1510.2 (1073.6 - 1978.8) | 67.5  (35.1-179.1) | 77.5  (25.2-174.4) |
| **T_max_ (hr)** | 4.0  (2.0 - 6.0) | 3.0  (2.0 - 6.0) | 6.0  (3.0 - 12.0) | 4.0  (3.0 - 6.0) | 6.0  (4.0 - 24.0) | 6 .0  (3.0 - 8.0) | 4.0  (2.0 - 12.0) | 3.5  (1.0 - 6.0) | 6.0  (2.0-8.0) | 6.0  (6.0-12.0) |
| **t_1/2_ (hr)** | 9.2  (2-22.4) | **8.5**  **(1.4-17)** | 10  (4.8-15.4) | 8.9  (5.5-18.6) | 9.4  (5.9-15.6) | 10.7  (5-23.4) | 9.4  (7.4-11.7) | 9.7  (6.5-14.7) | 55  (25.6-97.8) | 39.2  (19.5-98.9) |
| **AUC_0-t_ (hr*ng/mL)** | 163.5  (20.9-665.2) | 178  (18-1461) | 6144.5 (2888.6-12298.9) | 7114.5 (2323.8-20029.9) | 325.3 (154.6-2679.4) | 403.8  (134.2-1483.4) | 24323.5 (15760.4-35088.1) | 23054.5 (15265.7-33674.2) | 1780.3 (557.5-2430.2) | 1905 (684.9-5424.9) |
| **AUC_0-INF_(hr*ng/mL)** | 175.9  (22.9-666.4) | 179.7  (18.2-1328.6 | 6190.1 (2924.1-12471.4) | 7730.9 (2331.1-20389.8) | 332.9  (158-2723) | 412.8  (137.6-1491.8) | 24378.8 (15794.5-35171.7) | 23164.8 (15305.4-34987.2) | 2070.6 (593.6-3050.4) | 1896 (714.5-5824.5) |
| **V_z/F_ (L)** | 23044  (5449.4-350814.1) | 22874.5 (3450.9-402663.8 | 919.3  (424.2-2672) | 644.4 (257.7-2455.6) | 15957.2 (1994.2-34250.7) | 15687  (3593.8-58789.8) | 106  (84.8-170.8) | 115.8  (81.2-165.5) | 498.9 (225.4-1661.8) | 393.1 (122.1-1025.9) |
| **Cl/F (L/hr)** | 2277.9 (600.2-17451.9) | 2226.5 (301.1-21951.7 | 64.6  (32.1-136.8) | 51.9  (19.6-171.6) | 1201.8 (146.9-2531.9) | 972.1  (268.1-2907.3) | 8.3  (5.3-11.6) | 8  (5.1-13.3) | 6.3  (3.9-20.2) | 7.1  (2.1-16.8) |
| **C_max_ adjusted to dose (ng/mL)** | 21.6  (1.9-113.3) | 27.0  (1.6-262.7 | 278.5  (137.6-561.5) | 331.6 (118.4-1256) | 16.3 (7.9-42.2) | 18.4  (7.1-114.2) | 1498 (1226.3-2043.2) | 1484.8 (1012.4-2233.4) | 64.9  (37.9-156.4) | 72.5  (25.2-172.9) |
| **AUC_0-t_ adjusted to dose (hr*ng/mL)** | 105.5  (12.5-452.4) | 109  (11.5-1194.2 | 4196.7 (1733.1-8324.2) | 4351.9 (1347.8-17957.6) | 219.8 (100.5-1580.9) | 246.5 (77.8-1335) | 24540.5 (15924.1-33502.6) | 23220 (14395.9-44627.9) | 1665.7 (557.5-2590) | 1745.5 (662.1-5018) |
| **AUC_0-INF_ adjusted to dose(hr*ng/mL)** | 127.7  (13.8-453.4) | 111.6  (11.7-1195.7 | 4239.8 (1754.5-8419.7) | 4682.5 (1352-18107.6) | 238.9 (102.7-1606.6) | 255.4 (79.8-1342.6) | 24609 (15958.5-33582.4) | 23411.1 (14433.4-45028.3) | 1913 (593.6-3050.4) | 1731.7 (690.7-5387.7) |
