# Supplementary material for: Pharmacokinetics, safety, and efficacy of a single co-administered dose of diethylcarbamazine, albendazole and ivermectin in adults with and without Wuchereria bancrofti infection in Côte d’Ivoire
Source: PLoS Negl Trop Dis. 2019 May 20;13(5):e0007325. doi: 10.1371/journal.pntd.0007325 (PMC6550417; doi:10.1371/journal.pntd.0007325)
Supplement: S3 Table — (DOCX) [file pntd.0007325.s004.docx]

**Supplementary Table S3.** Non-compartmental pharmacokinetics estimates for ALB, ALB-OX, ALB-ON, DEC and IVM stratified by sex, men (N=32) and women (n=24) adults participants after received a single combined dose of ALB (400mg), DEC (12mg/kg) and IVM (0.2mg/kg, Median (range).

| **PK Parameters (Units)** | **Median (Range)** | | | | | | | | | |
| --- | --- | --- | --- | --- | --- | --- | --- | --- | --- | --- |
|  | **ALB** | | **ALB-OX** | | **ALB-ON** | | **DEC** | | **IVM** | |
|  | Male | Female | Male | Female | Male | Female | Male | Female | Male | Female |
| **C_max_ (ng/mL)** | 33.5  (2.7-336.0) | 33.8  (3.1-291.9) | 404.3  (209.4-1184.7) | 585.0 (204.2-1398.2) | 25.7  (12.2-71.1) | 31.0  (12.2-126.9) | 1494.0 (1073.6-1800.1) | 1703.4 (1262.6-2139.9) | 72.0  (34.0-174.4) | 74.2  (25.2-179.1) |
| **T_max_ (hr)** | 3.0  (2.0-6.0) | 4.0  (2.0-6.0) | 4.0  (3.0-12.0) | 6.0  (3.0-6.0) | 6.0  (3.0-24.0) | 6.0  (4.0-8.0) | 4.0  (1.0-12.0) | 4.0  (2.0-4.0) | 6.0  (2.0-8.0) | 6.0  (2.0-12.0) |
| **t_1/2_ (hr)** | 8.9  (1.4-17.8) | 9.0  (2.0-22.4) | 8.9  (6.2-18.6) | 9.4  (4.8-15.2) | 9.7  (5.0-23.4) | 10.9  (5.9-23.0) | 10.0  (7.4-14.7) | 8.6  (6.5-12.4) | 38.4  (19.5-96.0) | 56.0  (25.2-98.9) |
| **AUC_0-t_ (hr*ng/mL)** | 180.2  (18.0-1461.0) | 168.5  (20.6-1326.8) | 5953.1 (2713.4-20029.9) | 8170.6 (2323.8-19193.6) | 331.6 (157.6-2679.4) | 433.3  (134.2-1483.4) | 25036.9 (15265.7-33674.2) | 23207.3 (15760.4-35088.1) | 1909.0 (873.9-5424.9) | 1733.6 (557.5-3525.0) |
| **AUC_0-INF_(hr*ng/mL)** | 174.4  (18.2-1128.6) | 206.5  (21.3-1328.6) | 6190.1 (2727.4-20389.8) | 8218.0 (2331.1-19270.7) | 345.7 (173.4-2723.0) | 463.8  (137.6-1491.8) | 25165.1 (15305.4-34987.2) | 23253.6 (15794.5-35171.7) | 1969.3 (944.6-5824.5) | 2006.4 (593.6-3237.6) |
| **V_z/F_ (L)** | 25922.6  (3450.9-402663.8) | 19077.3 (3482.2-350814.1) | 932.2  (336.8-2483.1) | 606.3 (257.7-2672.0) | 16095.7 (1994.2-58789.8) | 13817.8 (3593.8-27320.8) | 115.2 (82.5-170.8) | 106.8  (81.2-143.1) | 383.6 (122.1-1192.0) | 563.2 (242.6-1661.8) |
| **Cl/F (L/hr)** | 2296.3  (354.4-21951.7) | 1972.2 (301.1-18800.6) | 64.6  (19.6-146.7) | 48.7  (20.8-171.6) | 1157.5 (146.9-2306.9) | 862.9  (268.1-2907.3) | 8.1  (5.1-13.3) | 8.1  (5.7-11.7) | 7.0  (2.1-14.3) | 6.4  (3.7-20.2) |
| **C_max_ adjusted to dose (ng/mL)** | 22.6  (1.6-199.9) | 21.8  (1.9-262.7) | 257.8  (125.6-704.9) | 331.6 (118.4-1256.0) | 16.1  (7.9-42.0) | 17.8  (7.1-114.2) | 1463.2 (1012.4-1888.5) | 1650.3 (1294.5-2233.4) | 70.1  (33.4-161.3) | 69.1  (25.2-172.9) |
| **AUC_0-t_ adjusted to dose (hr*ng/mL)** | 115.2  (11.5-869.3) | 96.0 (11.9-1194.2) | 3720.5 (1736.6-11917.8) | 4594.3 (1347.8-17957.6) | 223.0 (100.9-1580.9) | 236.1  (77.8-1335.0) | 25123.1 (14395.9-33456.8) | 22218.9 (15924.1-44627.9) | 1754.8 (853.4-5018.0) | 1561.7 (557. 5-3642.5) |
| **AUC_0-INF_ adjusted to dose(hr*ng/mL)** | 116.7  (11.7-829.5) | 135.2  (12.3-1195.7) | 4059.0  (1745.6-12131.9) | 4604.4 (1352.0-18107.6) | 227.9  (111.0-1606.6) | 264.1  (79.8-1342.6) | 25269.9 (14433.4-34761.4) | 22281.9 (15958.6-45028.3) | 1891.7 (1002.0-5387.7) | 1828.2 (593.6-3183.6) |
